# Beyond black boxes: using explainable causal artificial intelligence to separate signal from noise in pharmacovigilance

**DOI:** 10.1007/s11096-025-02004-z

**Published:** 2025-09-01

**Authors:** Renato Ferreira-da-Silva, Ricardo Cruz-Correia, Inês Ribeiro

**Affiliations:** 1 Porto Pharmacovigilance Centre, Faculty of Medicine of the University of Porto, Alameda Professor Hernâni Monteiro, 4200-319 Porto, Portugal; 2 RISE-Health, Department of Community Medicine, Information and Health Decision Sciences, Faculty of Medicine of the University of Porto, Porto, Portugal

**Keywords:** Pharmacovigilance, Drug-related side effects and adverse reactions, Artificial intelligence, Machine learning, Causality, Digital health

## Abstract

Artificial intelligence (AI), particularly machine learning (ML), is increasingly influencing pharmacovigilance (PV) by improving case triage and signal detection. Several studies have reported encouraging performance, with high F1 scores and alignment with expert assessments, suggesting that AI tools can help prioritize reports and identify potential safety issues faster than manual review. However, integrating these tools into PV raises concerns. Most models are designed for prediction, not explanation, and operate as “black boxes,” offering limited insight into how decisions are made. This lack of transparency may undermine trust and clinical utility, especially in a domain where causality is central. Traditional ML relies on correlational patterns and may amplify biases inherent in spontaneous reporting systems, such as under-reporting, missing data, and confounding. Recent developments in explainable AI (XAI) and causal AI aim to address these issues by offering more interpretable and causally meaningful outputs, but their use in PV remains limited. These methods face challenges, including the need for robust data, the difficulty of defining ground truth for adverse drug reactions (ADRs), and the lack of standard validation frameworks. In this commentary, we explore the promise and pitfalls of AI in PV and argue for a shift toward causally informed, interpretable models grounded in epidemiological reasoning. We identify four priorities: incorporating causal inference into AI workflows; developing benchmark datasets to support transparent evaluation; ensuring model outputs align with clinical and regulatory logic; and upholding rigorous validation standards. The goal is not to replace expert judgment, but to enhance it with tools that are more transparent, reliable, and capable of separating true signals from noise. Moving toward explainable and causally robust AI is essential to ensure that its application in pharmacovigilance is both scientifically credible and ethically sound.

## Introduction: the causality challenge in pharmacovigilance

Pharmacovigilance (PV) – the science and activities related to detecting, assessing, understanding, and prevention of adverse effects or any other drug-related problems [1] – faces a longstanding challenge: distinguishing true causality from mere association. Adverse drug reactions (ADRs) are common, with a pooled prevalence of 8.3% in primary care (of which ~ 23% are considered preventable, 95% CI 12.4–38.1%) and affecting up to ~ 16.9% of hospitalised patients (6.7% serious, 0.3% fatal) [2, 3]. Many serious ADRs only emerge post-approval, as clinical trials often miss rare or delayed effects due to limited sample sizes and strict eligibility criteria [4]. Hence, post-marketing surveillance is crucial to detect harm signals missed pre-approval. Yet, inferring causality from real-world data (RWD) remains notoriously difficult. Spontaneous reporting systems (SRS), electronic health records (EHR), and other RWD sources yield numerous drug-event correlations, but these do not equate to causation [5, 6]. A reported pair may be coincidental or confounded by underlying conditions. Traditional PV relies on expert judgment and algorithms (e.g., Naranjo scale for case-by-case assessment, and Bradford Hill criteria more appropriately applied to aggregated data) for case-by-case causality assessment, but these are subjective, depend on the quality of the information available in the report and vary across assessors and products [7–9]. In aggregated data, disproportionality metrics (e.g., reporting odds ratios or information component) can flag associations, but without proper controls or design, SRS data alone cannot confirm true causal links [6]. Robust causality assessment often requires epidemiological studies or accumulated evidence – both time-consuming and resource-heavy. In sum, PV professionals face a deluge of associations but lack strong tools to discern truly causal drug-event links [10]. This gap between correlation and causation is where artificial intelligence (AI) – especially explainable and causal AI – is increasingly seen as a promising solution.

## Current AI applications in drug safety: promise and pitfalls

AI – especially machine learning (ML), a subset of AI that learns patterns from data without explicit programming – has increasingly shaped PV in recent years, from automating literature screening to supporting signal detection. Its main advantage is speed and scalability: algorithms can process large volumes of data (e.g., VigiBase ADR reports, clinical notes, insurance claims) much faster than humans, identifying safety signals. Since 2019, there has been a marked increase in the use of ML for ADR detection from RWD [11]. Models using techniques like random forests, gradient boosting, and neural networks have been developed to classify reports or predict which patients may experience ADR [11]. The F1 score – the harmonic mean of precision (the proportion of flagged reports that are truly informative) and recall (the proportion of all informative reports correctly identified), ranging from 0 to 1 with 1 indicating perfect precision and recall – is commonly used to evaluate such models. Some classifiers distinguish ‘informative’ adverse event reports – those containing adequate clinical and temporal detail for causality assessment – from less useful ones, achieving over 80% accuracy based on the F1 score [12]. Other models predict whether an individual case safety report meets a minimum causality threshold (e.g., at least ‘Possible’ ADR); in this context, an FDA study evaluating ML models for ICSR prioritization reported F1 scores above 0.75, reflecting a good balance of precision and recall in identifying such cases [12]. At the case level, Cherkas et al. developed a model that aligned with expert causality assessments in 77–78% of drug-event pairs [13]. These examples show that while AI can enhance signal detection, case triage, and consistency – offering operational gains even in a traditionally expert-driven field – their contribution to true causality assessment remains limited, as most models still prioritise association over causal inference.

Yet, significant pitfalls limit current AI approaches in PV. Most AI models used so far are “black boxes” focusing on correlation rather than true causation. They excel at predicting outcomes but not at explaining them [14, 15]. A deep learning model might flag a statistical association between a drug and an outcome, but it offers no insight into why the link exists (or whether it is spurious). As Zhao et al. note, the goals of prediction vs. explanation diverge: model weights or feature importances are not equivalent to causal effect sizes [15]. In other words, an ML model might heavily weight a feature (e.g., concomitant use of Drug B) as a predictor of ADR risk for Drug A, but this could reflect confounding (patients on Drug A also take Drug B, which actually causes the event) rather than a direct causal role of Drug A. Without caution, AI may learn biases present in the data – for example, under-reporting and channeling biases in SRS can lead to distorted associations [6, 16]. Moreover, different data sources have intrinsic biases: an EHR-derived dataset might systematically miss non-serious events, while social media data might over-represent certain patient demographics. Current AI algorithms do little to address these confounders; they often amplify them since they optimize purely for predictive accuracy. This raises a trust problem: regulators and clinicians are understandably hesitant to act on an AI-generated signal without a clear rationale. If an algorithm flags Drug X for liver injury risk but cannot explain that decision, PV experts must still undertake a manual investigation to verify causality – negating much of the AI’s supposed efficiency gain. In short, the *status quo* of AI in PV provides speed, but not necessarily insight.

### Explainable AI and causal AI: concepts and potential in PV

Enter explainable AI (XAI) and causal AI – two complementary approaches designed to bridge gaps in interpretability and causality. Explainable AI refers to methods that make a model’s decisions transparent and understandable to humans [17]. Unlike “black box” models, XAI algorithms provide reasoning or highlight key features behind a prediction. In drug safety, this is particularly important: healthcare decisions require clarity on which factors triggered an alert, ideally aligning with known pharmacological or patient risk factors [18].

Some ML models are inherently more explainable (decision trees, rule-based learners), and commonly used techniques like SHAP or LIME can provide post-hoc explanations even for complex models by indicating each feature’s contribution to a particular prediction. However, it is vital to note that explanation does not necessarily imply causation. Holzinger et al. emphasize the concept of “causability” – the user’s ability to understand and trust AI-generated explanations – and argue that conventional XAI methods typically highlight statistical associations rather than genuine causal processes [19]. For example, a random forest model might identify “polypharmacy” and “advanced age” as top predictors of an ADR, but without rigorous causal analysis, these variables might simply serve as proxies for other unmeasured factors. Thus, while XAI can suggest plausible decision pathways, it does not offer definitive proof of causality [19]. Indeed, some researchers warn that current XAI methods can create a false sense of security. Ghassemi et al. argued that today’s popular explainability tools often fail to deliver true transparency or safety, calling reliance on them a “false hope” in healthcare [20]. In other words, showing a heatmap or feature weight might placate our need for an explanation, while the real logic of a deep model remains opaque. This critique urges that while explainability is important, it must be paired with rigorous validation – much like we trust a drug’s clinical efficacy even if its mechanism isn’t fully understood [20].

Causal AI, by contrast, seeks to model cause-and-effect rather than mere correlation, drawing on frameworks from epidemiology and causal inference – such as causal graphs, do-calculus, and counterfactual reasoning – within AI systems. The goal is to embed domain knowledge and causal assumptions so that outputs better address “Did drug X cause outcome Y?” rather than just “Is X associated with Y?”. In PV, this is both promising and complex. On one hand, even basic causal inference by AI could help prioritise signals more intelligently, focusing on those more likely to reflect true ADRs, and potentially reveal causal links faster. On the other hand, causal inference demands robust data and careful design – rarely present in noisy PV datasets.

A recent scoping review found that despite the surge in AI for PV, explainable and causal AI techniques remain underexplored in this domain [11], highlighting a ripe opportunity. A few pioneering efforts demonstrate what might be possible. Wang et al. introduced InferBERT, a novel framework integrating a transformer-based NLP model with Judea Pearl’s do-calculus for causal inference [21]. By analyzing FAERS case narratives, their model could not only predict outcomes but also infer likely causal factors; for example, it identified key drug-related causes for acute liver failure and organized them into a causal tree [21]. In case studies, InferBERT achieved impressive accuracy (around 78%-95%) in classifying which cases of analgesics-related acute liver failure and tramadol-related mortalities were truly caused by the drug (versus coincidental) [21]. Moreover, the inferred causal factors aligned well with clinical knowledge, suggesting the approach wasn’t merely fitting noise. This is an exciting proof-of-concept that AI can incorporate causal reasoning to some degree, providing outputs that are both predictive and interpretable as causal insights. Similarly, other researchers have experimented with integrating causal algorithms into PV analytics. A recent study by Dimitsaki et al. applied causal deep learning (CDL) to detect ADR using EHR, focusing on drug-induced acute kidney injury as a case study [22]. Their approach demonstrated how explainable causal relationships could be mined from RWD, illustrating the potential of combining AI with formal causal inference to strengthen signal validation processes in PV[22]. Early studies like these remain scarce but illustrate the potential: causal-aware AI could triage signals with an extra layer of reasoning, focusing attention on patterns more likely to reflect true drug harms.

### Critical perspectives: limitations and opportunities

While XAI and causal AI offer a promising path forward, it is important to critically assess their limitations in the PV context. A central concern is that most XAI methods provide local explanations (e.g., why a model flagged a specific case) but do not guarantee global causal validity. An explanation might say a warfarin case was flagged due to “age > 70” and “concurrent NSAID use,” which are known risk factors for bleeding – a plausible rationale. However, if the underlying model is mis-specified or the data are biased, such explanations could be post hoc justifications rather than evidence of a truly causal signal. Moreover, PV datasets are full of noise and confounders that can trip up even formal causal inference methods. Causal AI frameworks typically require one to assume a causal graph or use instruments; if important confounders (like a patient’s comorbid conditions) are unmeasured or unknown, the results might be as unreliable as a naive correlation. Data quality is a recurring obstacle: high rates of under-reporting, missing data, and poorly recorded covariates plague spontaneous reports [6]. Integrating multiple data sources can help – for example, linking SRS with EHR data to obtain denominators or patient details – but this comes with technical and privacy complexities. Another challenge is validation: how do we evaluate a causal inference algorithm in PV? Unlike prediction tasks with clear accuracy metrics, causal conclusions are hard to benchmark outside of well-understood “truth” cases. We have very few ground truth datasets where we know a drug caused an ADR (and at what rate) to test these algorithms. Zhao et al. suggest developing benchmark datasets annotated with known causal relationships as one step to facilitate XAI/causal model development [15]. Additionally, the lack of human interpretability in causal models can be a barrier. Paradoxically, a sophisticated causal model might become a new kind of black box if its reasoning (say, a complex Bayesian network) isn’t easily interpretable by PV specialists.

## Final remarks and future directions

PV stands at a critical juncture, where the expanding availability of real-world data and the rapid evolution of AI make it increasingly feasible to transform how drug safety is assessed. To do so responsibly, a shift is needed from correlation-based detection to causally informed and interpretable approaches. Four priorities stand out:

I. Integrating traditional epidemiological designs – such as self-controlled case series, case–control designs, or propensity score–matched cohorts – into AI pipelines. For instance, algorithms could be trained within these frameworks so that temporal relationships, exposure definitions, and bias adjustment strategies are hard-coded into the model logic, combining bias control with algorithmic scalability. This could help bridge the gap between rapid automated detection and the methodological rigour required for causal inference in pharmacovigilance;
II. Establishing shared benchmarks for causal discovery in PV, ideally using curated, well-characterized datasets with historical safety signals and confirmed null associations. These benchmarks could be hosted in secure research environments and annotated by multidisciplinary panels, ensuring that both positive and negative controls are represented. Publicly available performance leaderboards could facilitate transparent evaluation and continuous improvement across research groups;
III. Fostering interdisciplinary collaboration between AI experts, pharmacologists, and epidemiologists to ensure outputs are clinically meaningful, interpretable, and aligned with causal reasoning frameworks (e.g., temporality, biological plausibility, coherence). Practical mechanisms could include standing advisory committees, structured joint audits of AI-generated signals, and simulation exercises to stress-test models before deployment in regulatory or clinical decision-making;
IV. Maintaining rigorous validation as a cornerstone, whereby AI-generated signals or causal inferences are only acted upon when supported by converging evidence—such as mechanistic plausibility, rechallenge data, dose–response relationships, or replication across independent data sources. This process could follow a tiered review system, beginning with automated plausibility checks, followed by structured human expert review, and culminating in formal regulatory or clinical assessment.

Ultimately, the promise of explainable and causal AI in PV lies not in replacing experts but in moving beyond black boxes – empowering decision-makers through models that can meaningfully distinguish true safety signals from background noise, while embedding clear operational pathways for their independent evaluation and confirmation, enabling faster, safer, and more causally grounded decision-making.

## Data Availability

No datasets were generated or analysed during the current study.
